# Supplementary material for: Structure of the DDB1-AMBRA1 E3 ligase receptor complex linked to cell cycle regulation
Source: Nat Commun. 2023 Nov 22;14:7631. doi: 10.1038/s41467-023-43174-6 (PMC10665379; doi:10.1038/s41467-023-43174-6)
Supplement: Supplementary file 1 — Supplementary file [file 41467_2023_43174_MOESM1_ESM.pdf]

Structure of the DDB1-AMBRA1 E3 ligase receptor complex linked to cell cycle regulation

Ming Liu[1], Yang Wang[1], Fei Teng[1,2], Xinyi Mai[1], Xi Wang[1], Ming-Yuan Su[2,3,4*], Goran Stjepanovic[1*]

1. Kobilka Institute of Innovative Drug Discovery, School of Medicine, The Chinese University of Hong Kong, Shenzhen, Shenzhen 518172, China
2. Department of Biochemistry, School of Medicine, Southern University of Science and Technology, Shenzhen 518055, China
3. Key University Laboratory of Metabolism and Health of Guangdong, Southern University of Science and Technology, Shenzhen 518055, China
4. Institute for Biological Electron Microscopy, Southern University of Science and Technology, Shenzhen 518055, China

*Correspondence: Ming-Yuan Su (sumy@sustech.edu.cn) and Goran Stjepanovic (goranstjepanovic@cuhk.edu.cn)

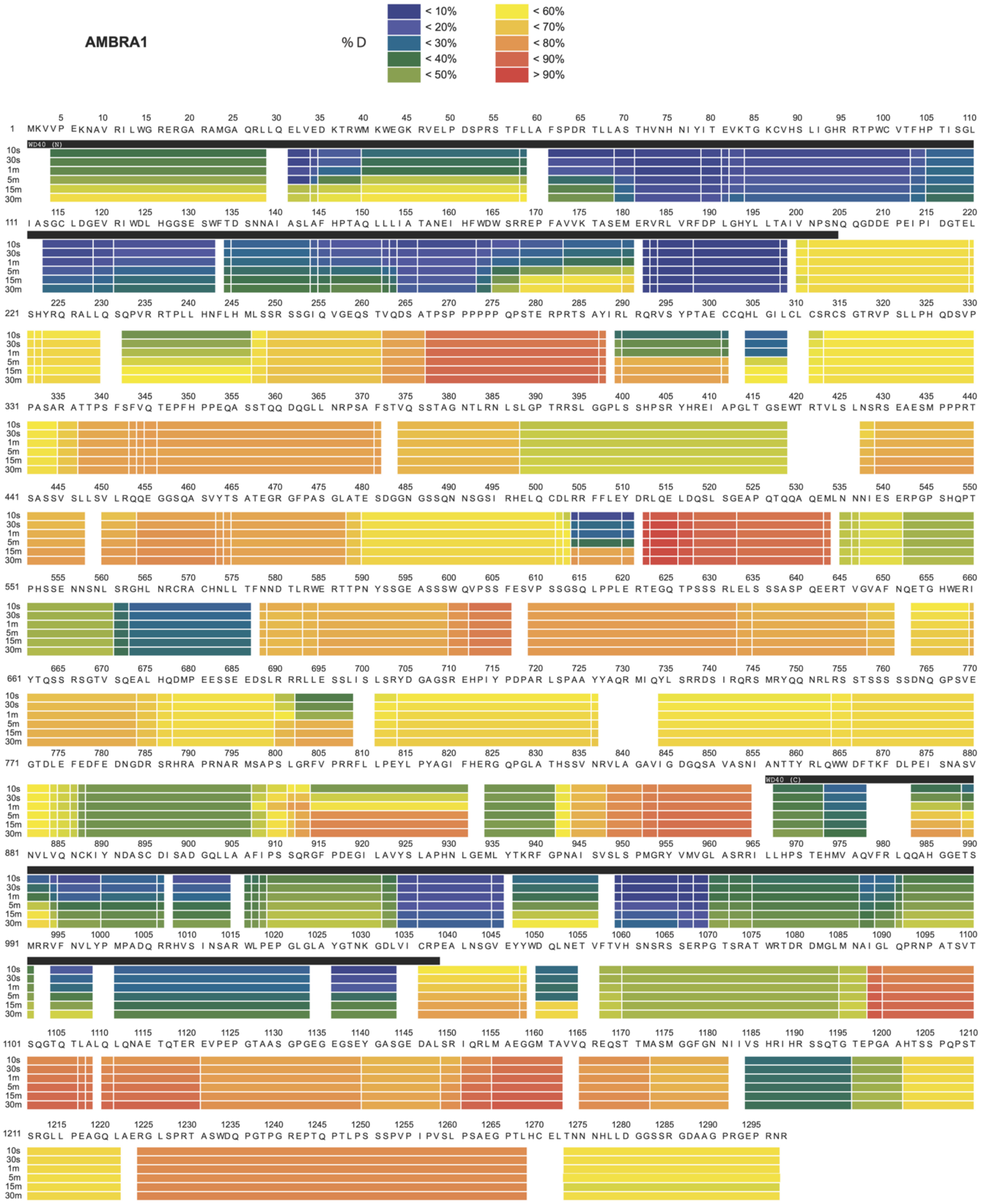

Supplementary Fig.1: Deuterium uptake data for full length AMBRA1.
HDX-MS data are presented in heatmap format. Absolute deuterium uptake after 10 s, 30 s, 1 m, 5 m, 15 m and 30 m are indicated by a color gradient below the protein sequences. WD40-N and WD40-C regions are indicated above the protein sequence.

a

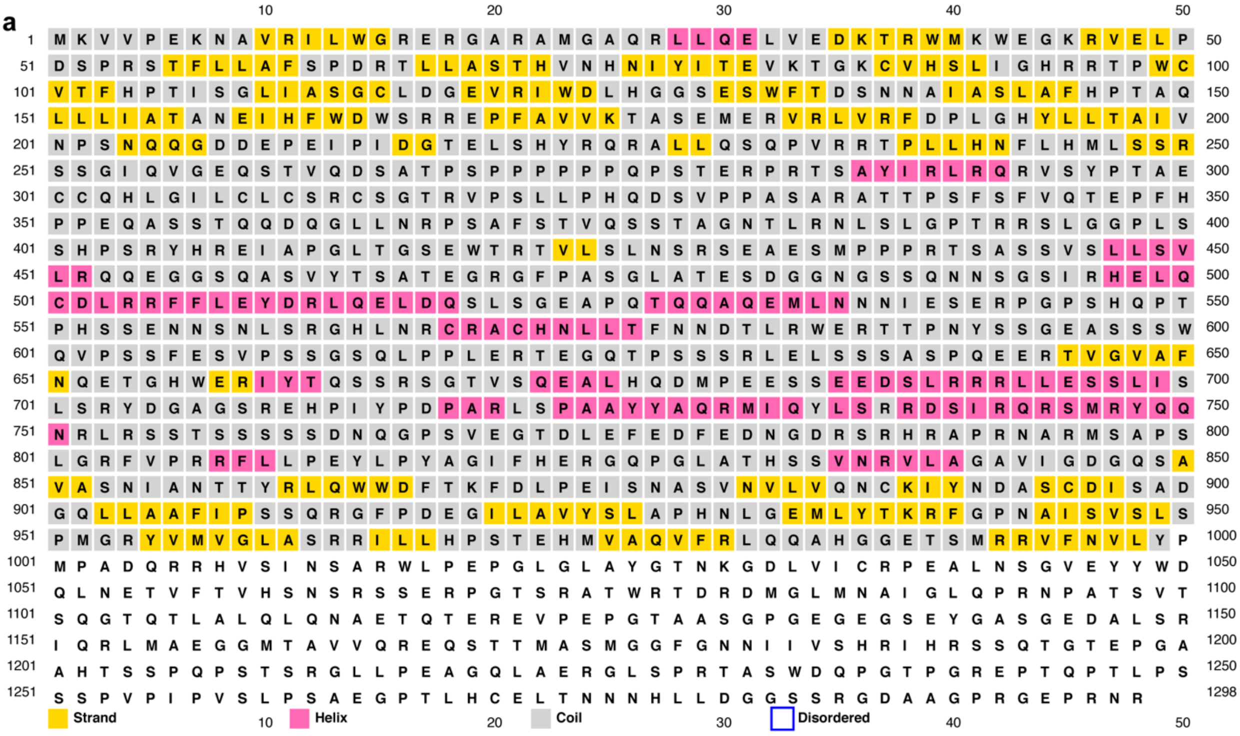

b

Repeat ID	Score*	Start	End	Strand_d	Loop_da	Strand_a	Loop_ab	Strand_b	Loop_bc	Strand_c	Loop_cd	H_bonds	Hotspots
1	63	44	82	GKRVEL	PDSPRS	TFLLAF	SPDRT	LLASTH	VNH	NIYITE	VKTG		F57
2	128	86	125	KCVHSL	IGHRRT	PWCVTF	HPTISG	LIASGC	LDG	EVRIWD	LHG	Tetrad	L116, W99
3	81	128	165	GSESWF	TDSNNA	IASLAF	HPTAQ	LLLIAT	AN	EIHFWD	WSRREP		
4	46	171	225	FAVVKT	ASEMER	VRLVRF	DPLGH	YLLTAI	VNPSNQQGDDEPEIPIDGT	ELSHYR	####		R182, R184

Supplementary Fig.2: Secondary structure and domain prediction of AMBRA1 by PSIPRED and WDSPdb2.0[1, 2].

a. Secondary structure annotation of AMBRA1. The protein secondary structure elements are shown in different colors (pink for α-helix, yellow for β-strands, and grey for coil).

b. WD40-repeat protein structure predictor (WDSP) prediction result for AMBRA1, indicating the first 225 residues harbor 4 WD40 repeats.

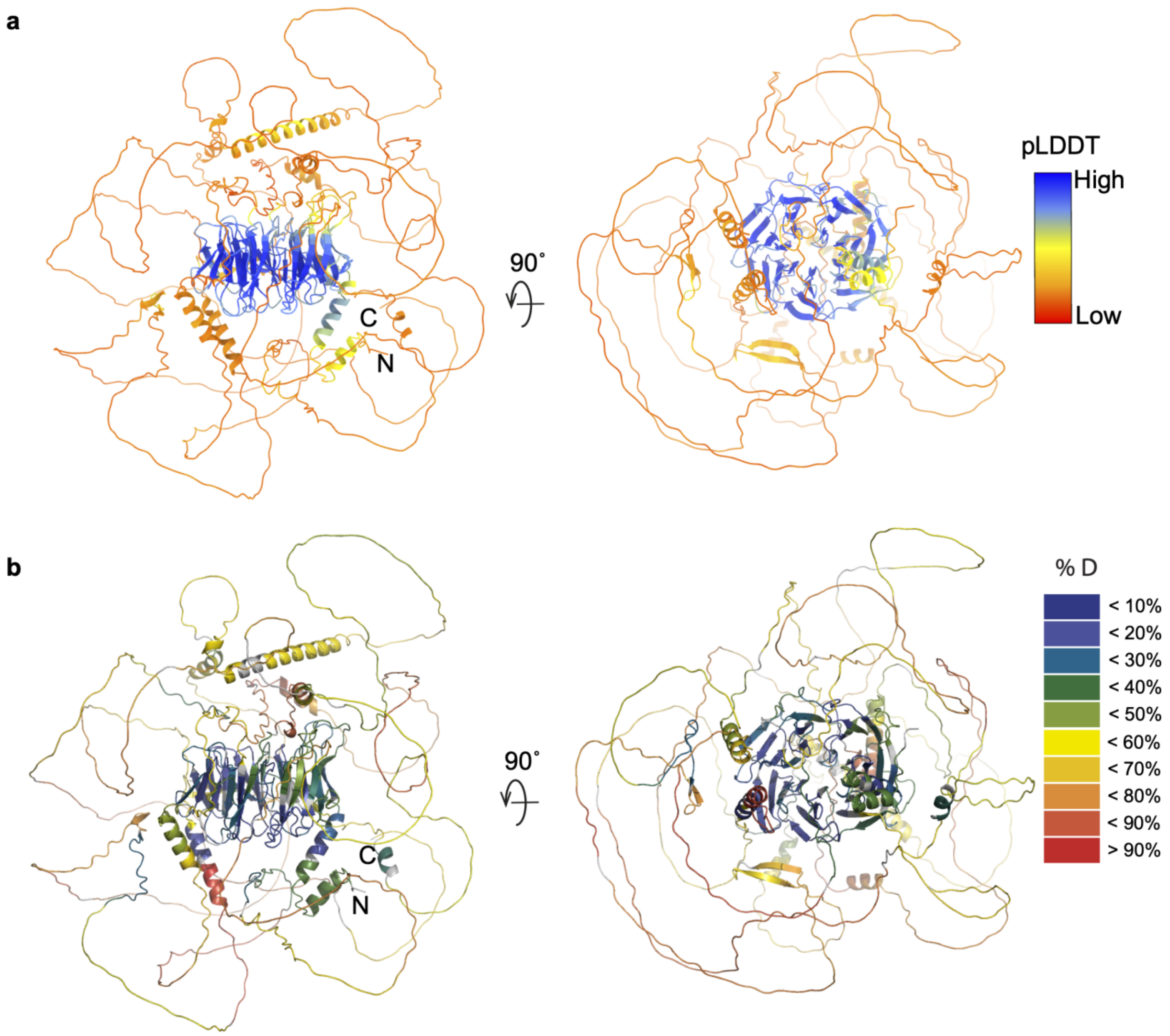

Supplementary Fig.3: Mapping of HDX-MS data to the Alphafold2 model of AMBRA1.

a. Alphafold2 colored by the confidence metric (pLDDT) of AMBRA1.

b. Absolute deuterium uptake after 10s is indicated using a blue-yellow-red gradient coloring scheme. Regions where no exchange data are available are colored gray.

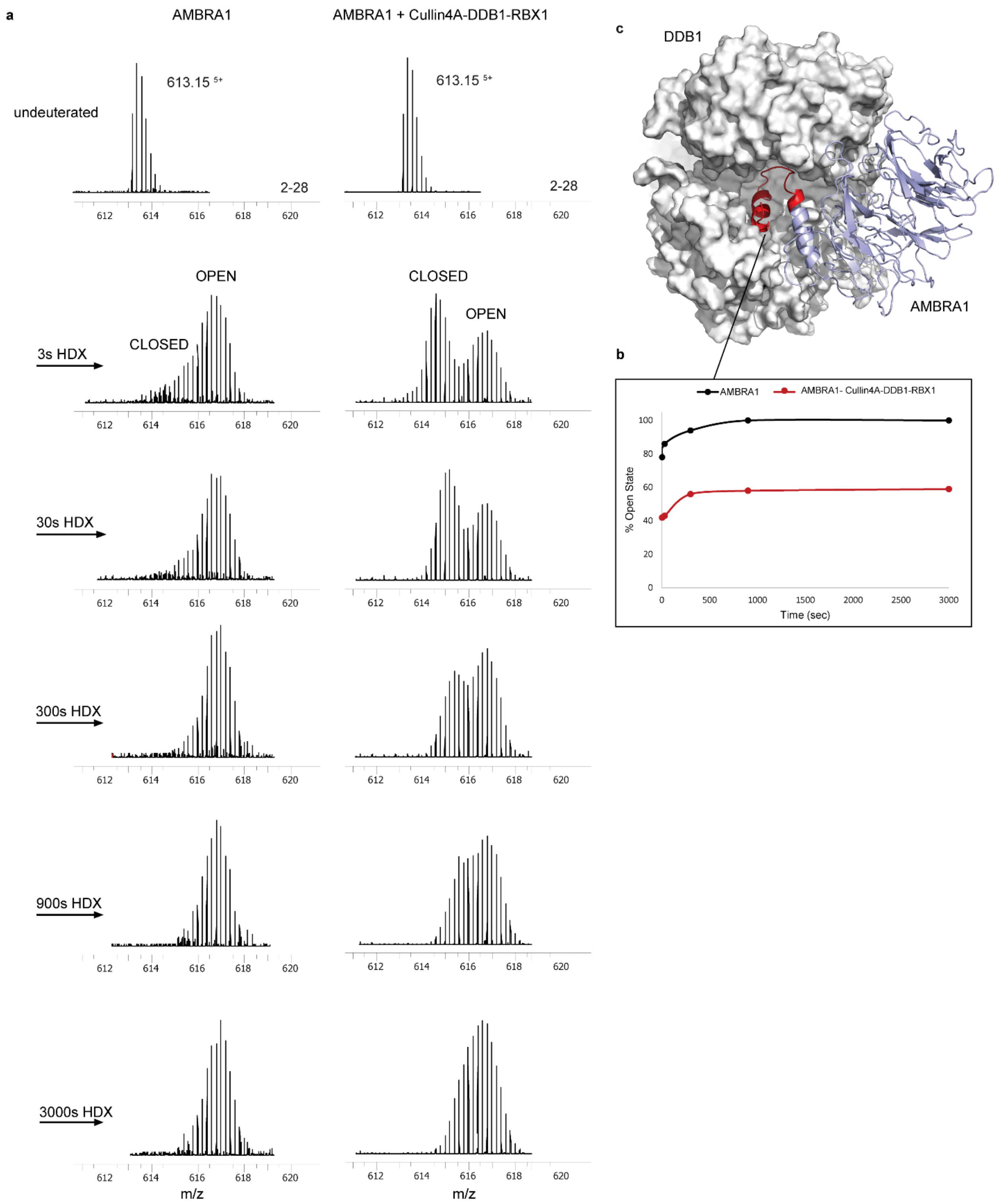

Supplementary Fig.4: Close to open transition kinetics for part of AMBRA1 N-terminal helix-loop-helix as seen by HDX.

a. Bimodal isotopic envelopes for the part of AMBRA1 N-terminal helix-loop-helix (peptide spanning residues 2-28), either alone or in the Cullin4A-DDB1-RBX1 E3 ligase complex. Deuteration time points are indicated. Close-to-open transition kinetics for the AMBRA1 helix-loop-helix motif are calculated by fitting two Gaussians to the high- and low-mass subpopulations.

b. Relative amount of the open state is plotted against deuteration time.

c. Structure of the AMBRA1-DDB1 complex interface showing the peptide spanning residues 2-28 colored in red. DDB1 is shown on the surface and colored gray, while AMBRA1 is colored blue.

WD40-N

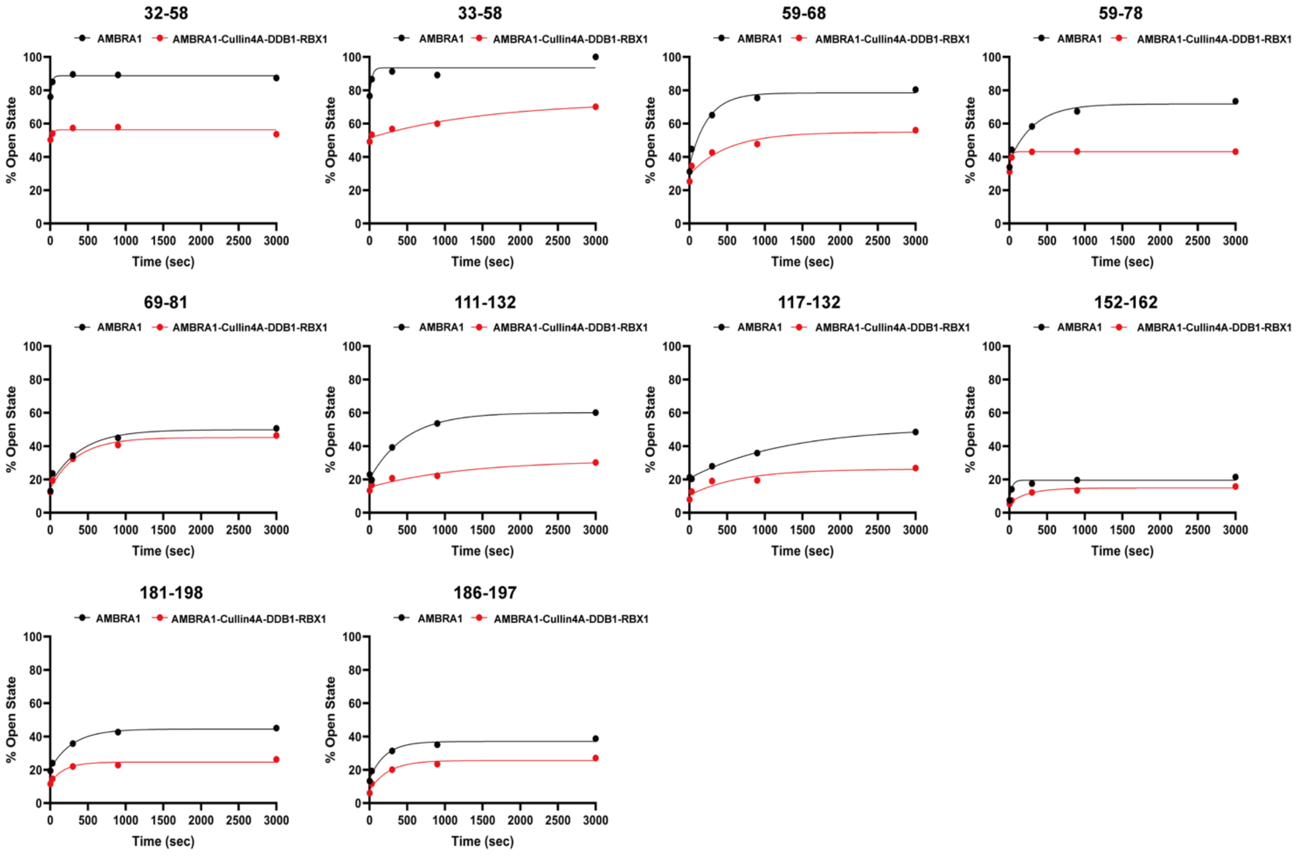
32-58
33-58
59-68
59-78
69-81
111-132
117-132
152-162
181-198
186-197
AMBRA1
AMBRA1-Cullin4A-DDB1-RBX1
% Open State
Time (sec)
0 500 1000 1500 2000 2500 3000
0 20 40 60 80 100

WD40-C

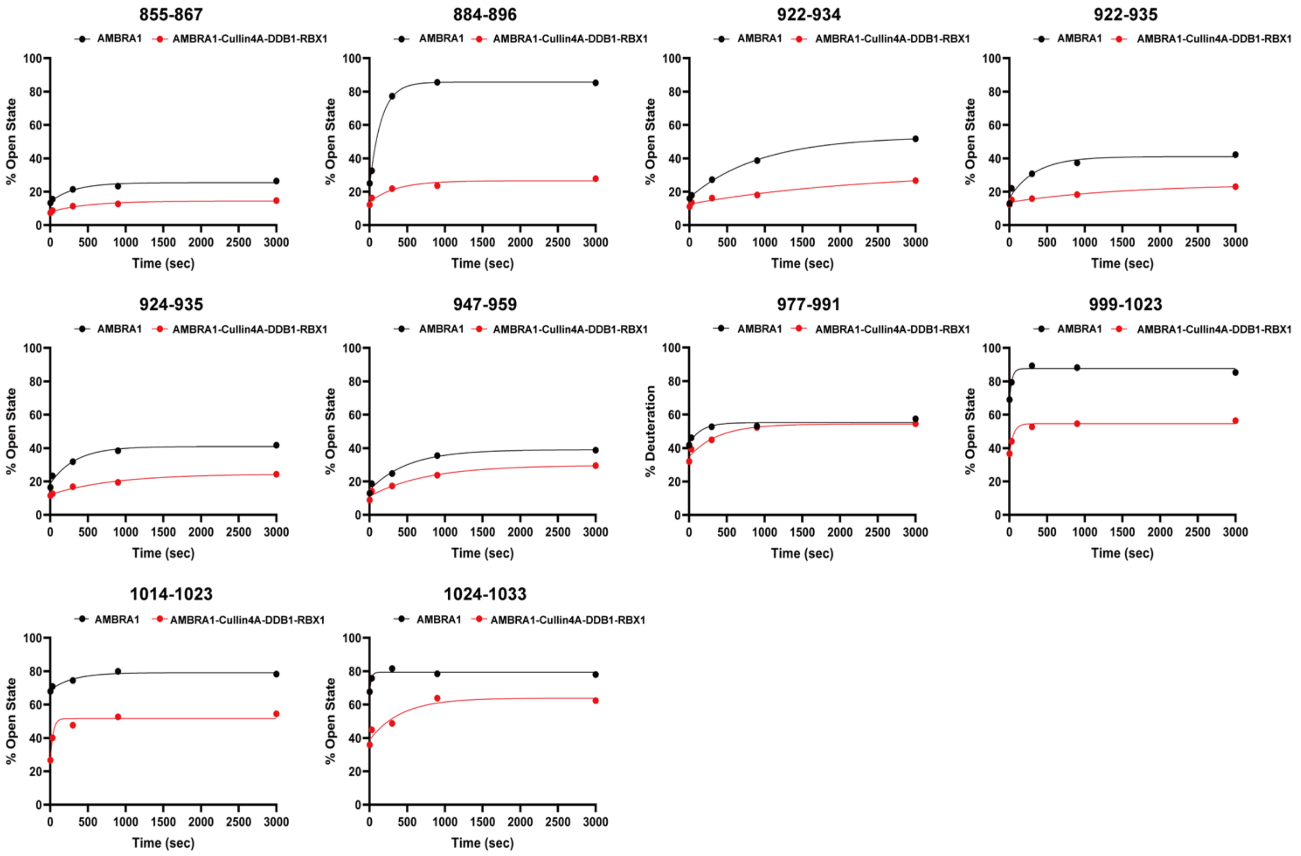
855-867
884-896
922-934
922-935
924-935
947-959
977-991
999-1023
1014-1023
1024-1033
AMBRA1
AMBRA1-Cullin4A-DDB1-RBX1
% Open State
% Deuteration
Time (sec)
0 500 1000 1500 2000 2500 3000
0 20 40 60 80 100

IDRs

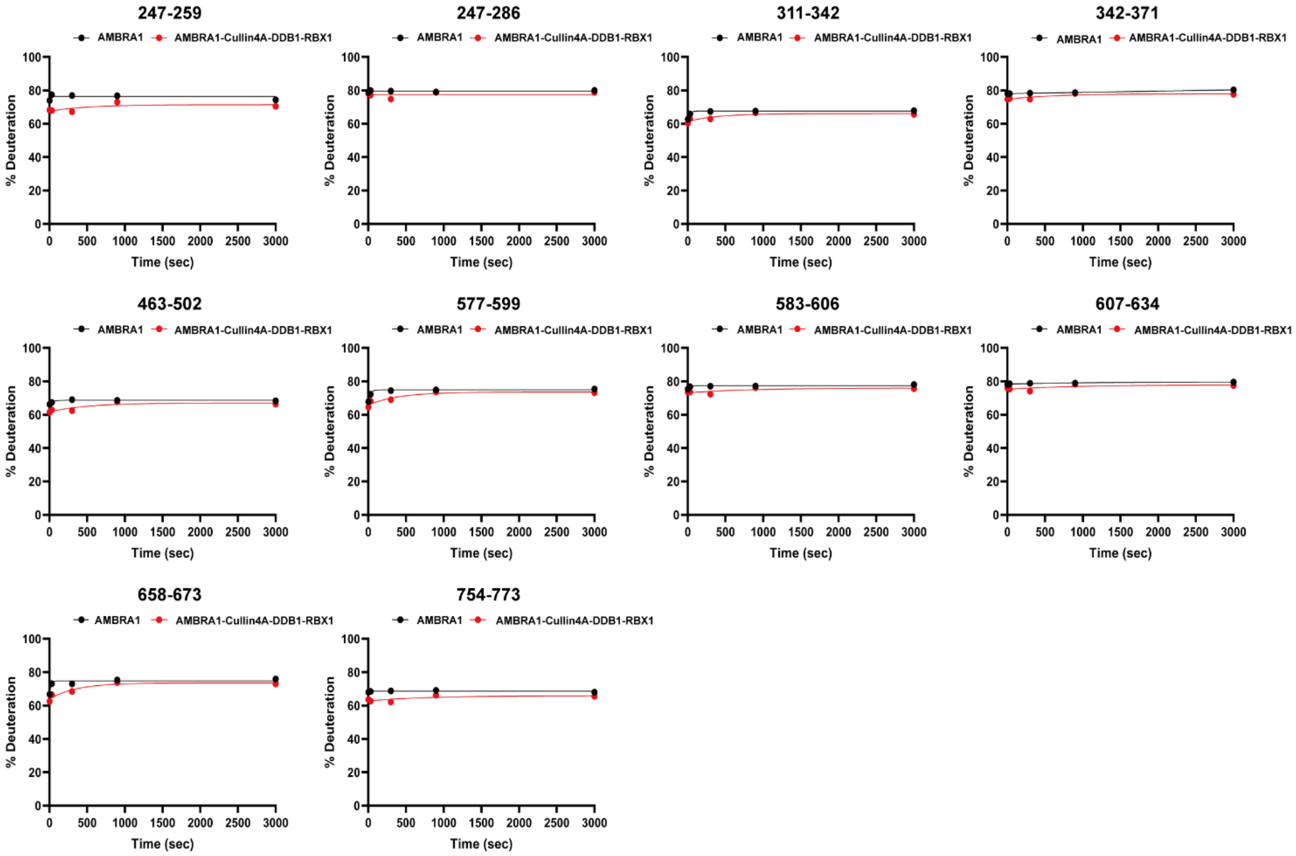

Supplementary Fig.5: HDX uptake plots for thirty AMBRA1 peptides in the presence (red) or absence (black) of Cullin4A-DDB1-RBX1 E3 ligase.
Deuterium exchange profiles of individual peptides from WD40-N, IDRs and WD40-C regions are shown. Residues covered by each peptide are shown at the top of each plot. Deuterium exchange is expressed as either % deuteration (peptides undergoing EX2 kinetics) or relative amount of the open state (peptides undergoing EX1 kinetics, also see Supplementary Fig. 4) and plotted against deuteration time. Each plot corresponds to measurements over five time points.

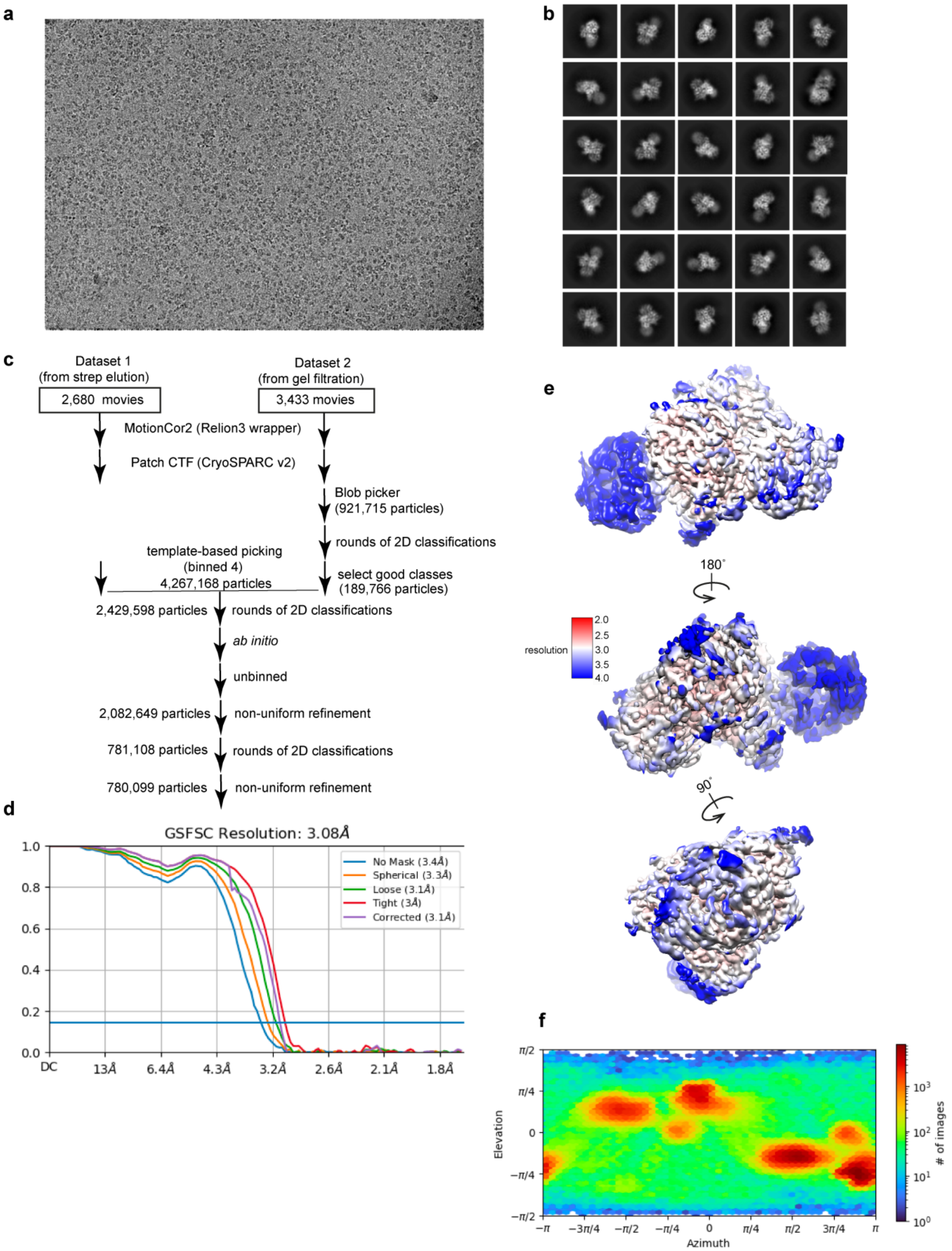

Supplementary Fig.6: Cryo-EM data process of the AMBRA1^{WD40} -DDB1 complex.

a. A representative motion-corrected cryo-EM micrograph of the AMBRA1^{WD40}-DDB1 complex.
b. Representative 2D class averages for the AMBRA1^{WD40}-DDB1 complex.
c. Flow chart of cryo-EM data processing.
d. The FSC plots are between two independently refined half-maps with no mask (blue), spherical mask (orange), loose mask (green), tight mask (red), and corrected (purple). A cut-off of 0.143 (blue line) was used to estimate the resolution.

e. Local resolution is colored as indicated in the scale.
f. Angular particle distribution calculated in cryoSPARC for particle projections. The heatmap shows the number of particles for each viewing angle.

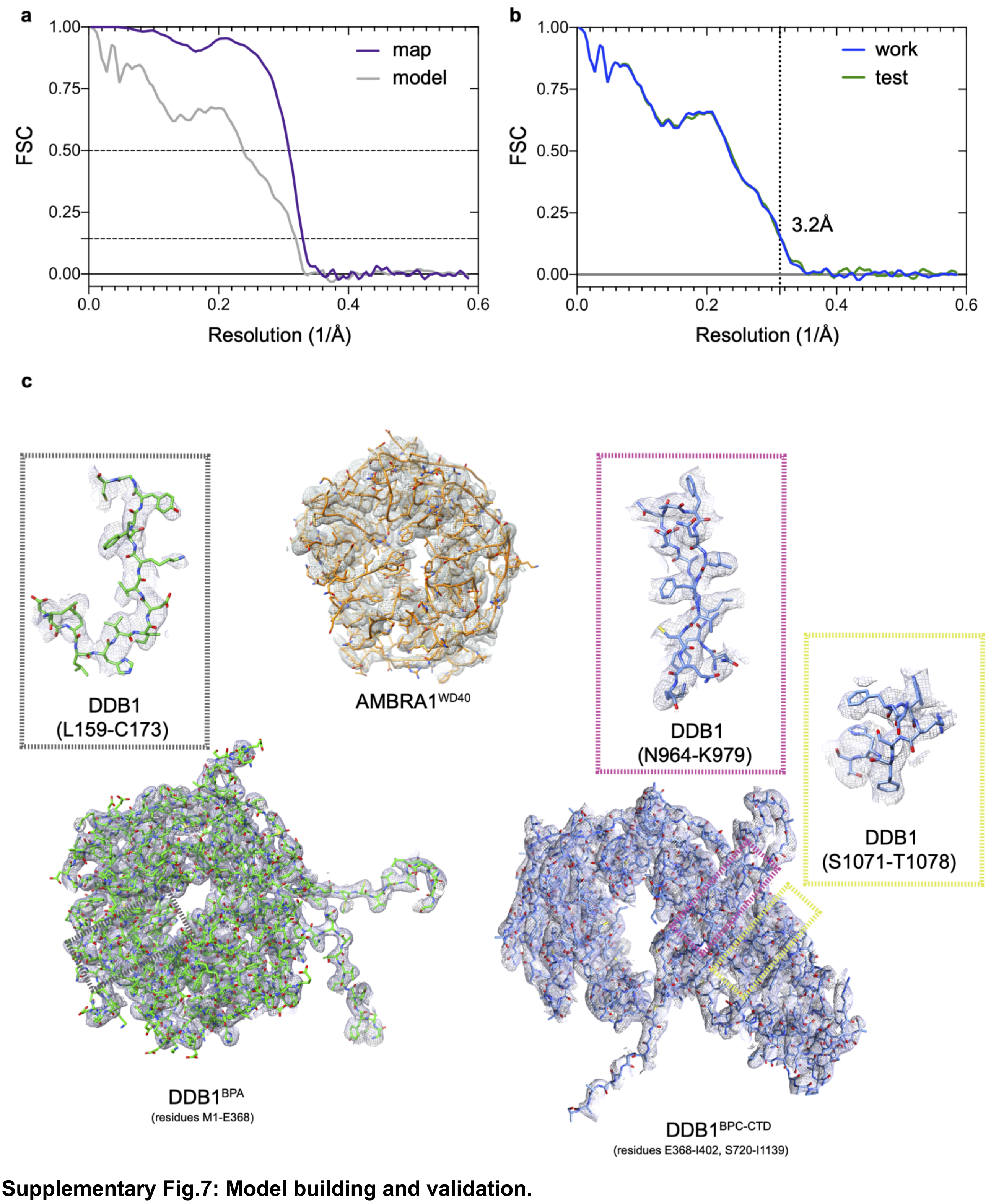

Supplementary Fig.7: Model building and validation.

a. Refinement and map-vs.-model FSC.
b. Cross-validation test FSC curves to assess overfitting. The refinement target resolution of 3.2 Å is indicated.
c. Refined coordinate model fit of the indicated region in the cryo-EM density. Zoomed-in views of the EM density (gray, pink and yellow meshes) for the domain structures.

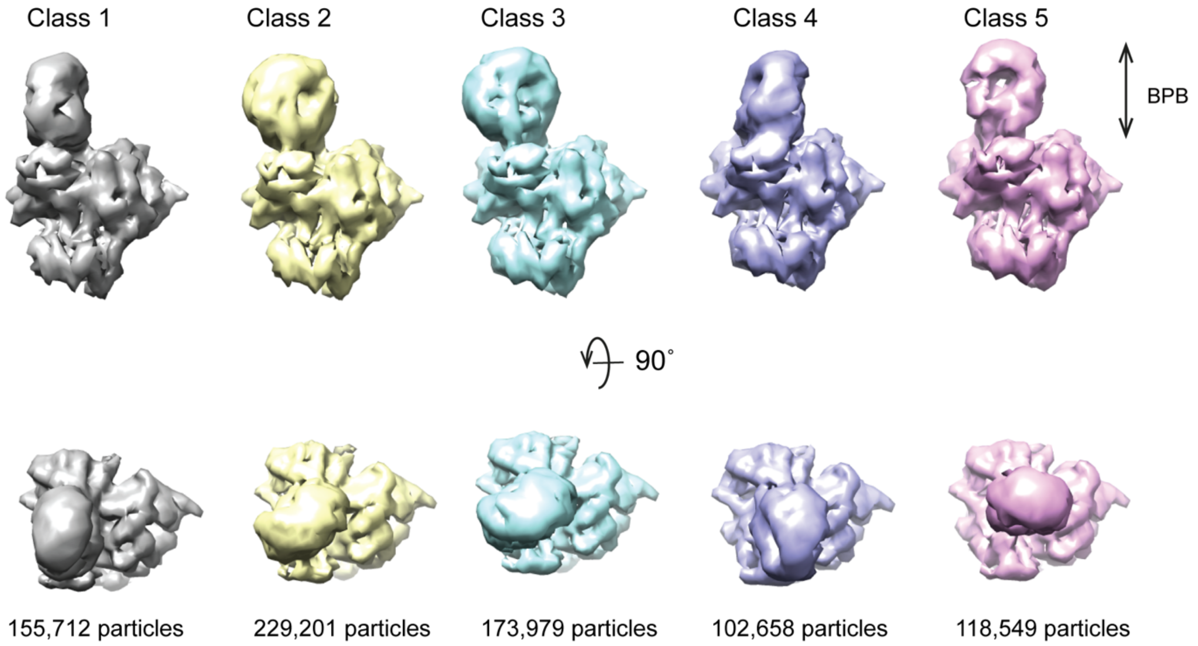

Supplementary Fig.8: Dynamics of the BPB domain of DDB1 that adopts different orientations relative to other domains.
780K refined particles from cryoSPARC were imported into Relion 3.1 for 3D classification without alignment analysis, resulting in 5 different classes presenting the dynamics of the BPB domain of DDB1.

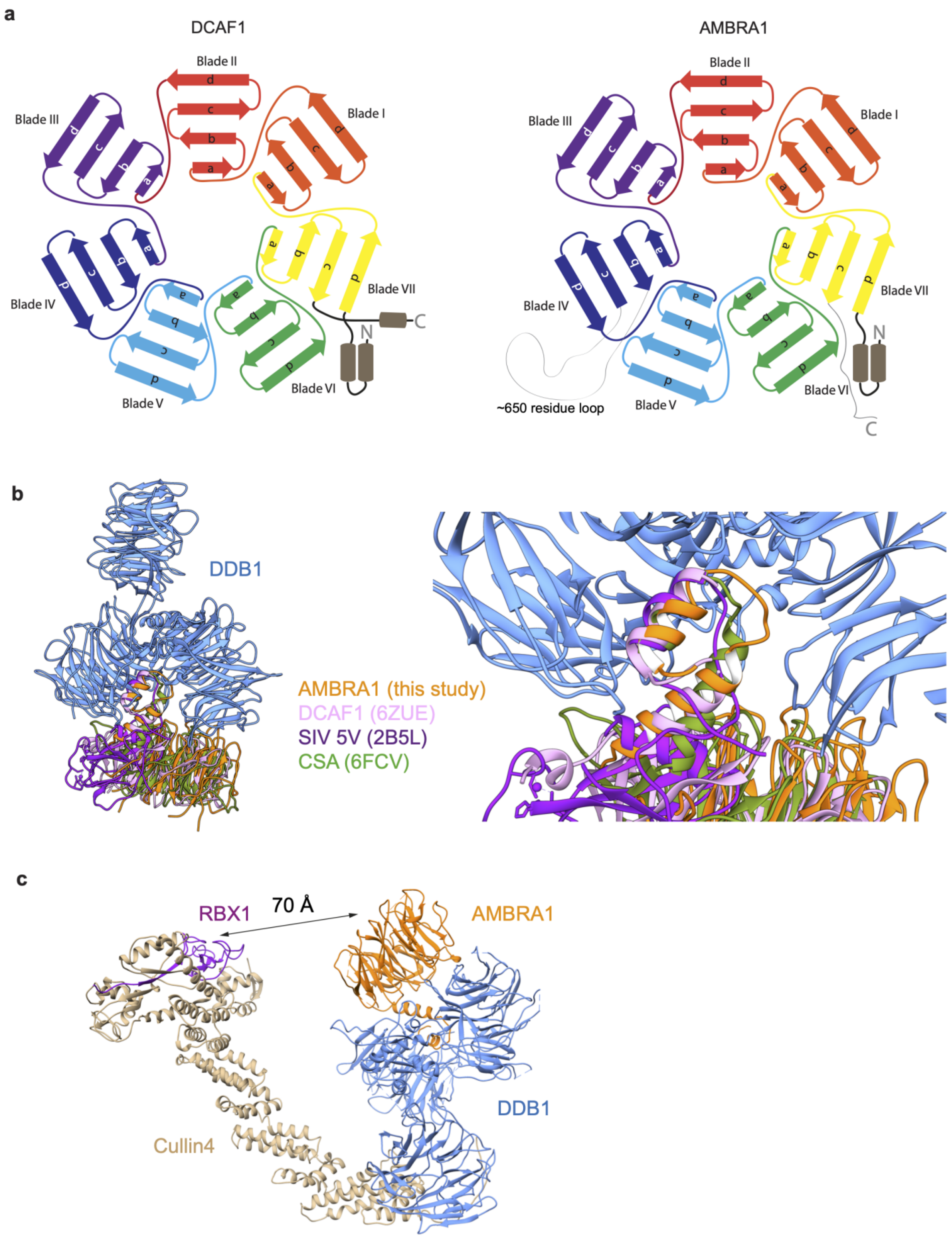

Supplementary Fig.9: Structural comparison between AMBRA1^{WD40} and other published DDB1-bound proteins.

a. Schematic diagram of the WD40 domain of DCAF1 (left) and AMBRA1 (right).
b. Structural comparison of AMBRA1 (colored in orange, this study), Simian virus 5V (colored in magenta, PDB 2B5L), DCAF1 (colored in pink, PDB 6ZUE) and CSA (colored in green, PDB 6FCV) bound to DDB1.
c. Model of the entire Cullin4-DDB1-RBX1-AMBRA1 E3 complex.

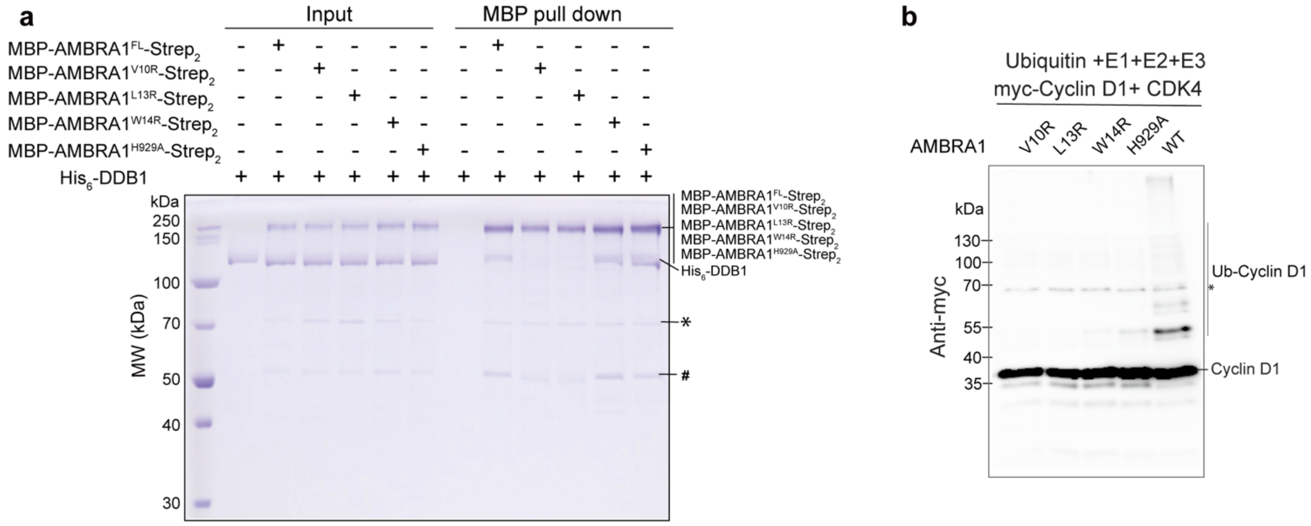

Supplementary Fig.10: Mutations in AMBRA1 disrupt the binding and ubiquitination of Cyclin D1.

a. *In vitro* pull-down experiment of the full-length and point mutants of AMBRA1 with His_6-tagged DDB1. The experiment was repeated at least three times and visualized by SDS–PAGE and Coomassie blue staining. Data presented is representative of n = 3 biological repeats. The asterisk and hash indicate hsp70 and other contaminations.
b. *In vitro* ubiquitination assay of AMBRA1 point mutants for Cyclin D1. Data presented is representative of n = 3 biological repeats. The asterisk indicates GST-myc-Cyclin D1.

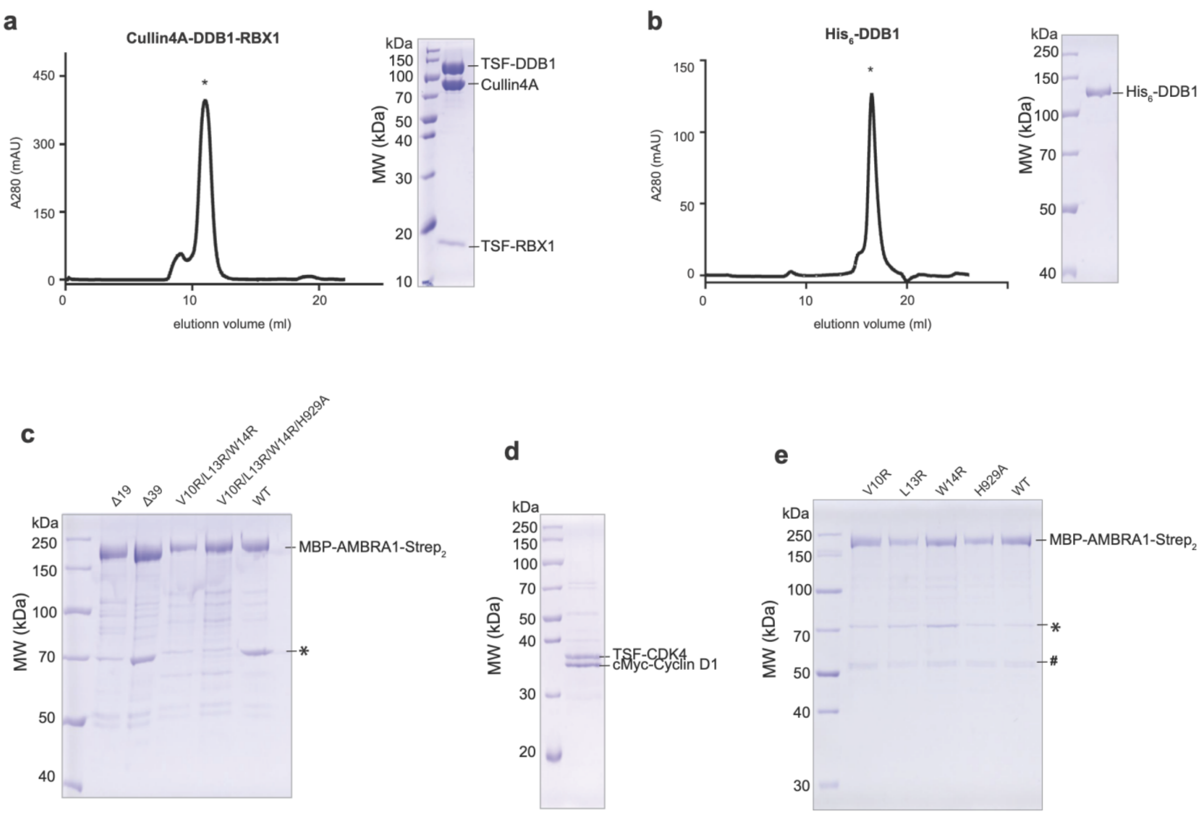

Supplementary Fig.11: Purification of Cullin4A-DDB1-RBX1 E3 ligase, DDB1, WT/truncated/mutated AMBRA1 and Cyclin D1-CDK4 complex.

a. The size exclusion profile (Superdex 200 Increase 10/300) and Coomassie blue-stained SDS–PAGE analysis of the purified protein Cullin4A-DDB1-RBX1(E3) ligase were used for the *in vitro* ubiquitination assay. mAU, milliabsorbance units.
b. The size exclusion profile (Superose 6 Increase 10/300) and Coomassie blue-stained SDS–PAGE analysis of DDB1, which was used for the MBP pull-down assay. mAU, milliabsorbance units.
c. Coomassie blue-stained SDS–PAGE analysis of WT/truncated/mutated AMBRA1. The asterisk indicates hsp70 contaminations.
d. Coomassie blue-stained SDS–PAGE analysis of the Cyclin D1-CDK4 complex.
e. Coomassie blue-stained SDS–PAGE analysis of the single mutated AMBRA1. The asterisk and hash indicate hsp70 and other contaminations.

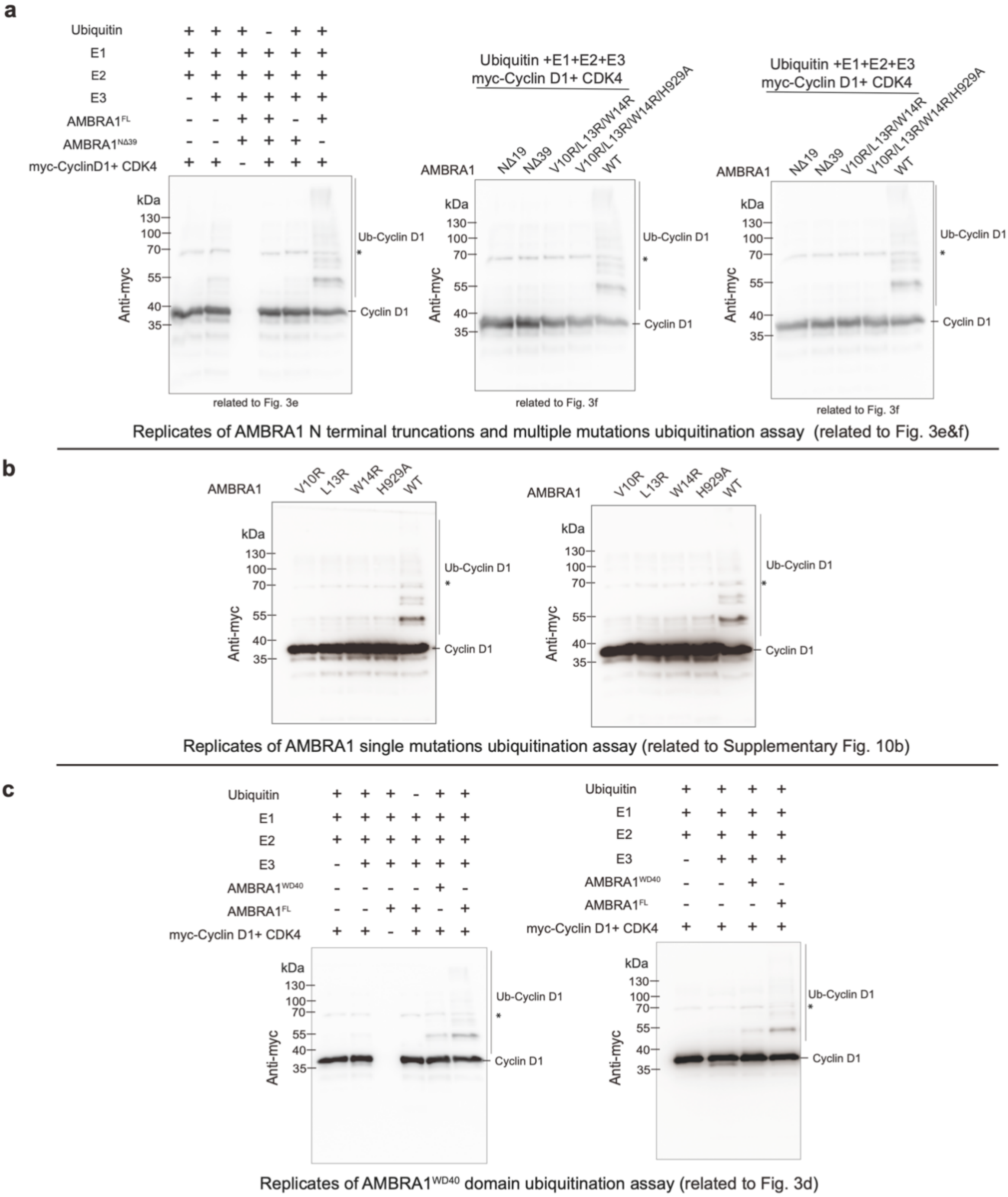

Supplementary Fig.12: Replicates of the *in vitro* ubiquitination experiments of full-length, N-terminal 39 residue-deleted, WD40 domain and mutated AMBRA1 for Cyclin D1. Ubiquitination assays were analyzed by SDS-PAGE, followed by immunoblotting. The experiment was repeated at least three times. The asterisk indicates GST-myc-Cyclin D1.

a. *In vitro* ubiquitination assay of the full-length, N-terminal 19 residue-deleted, N-terminal 39 residue-deleted and mutated AMBRA1 for Cyclin D1. Data presented are representative of n = 3 biological repeats.
b. *In vitro* ubiquitination assay of AMBRA1 point mutants for Cyclin D1. Data presented are representative of n = 3 biological repeats.
c. *In vitro* ubiquitination assay of AMBRA1^{WD40} domain for Cyclin D1. Data presented are representative of n = 3 biological repeats.

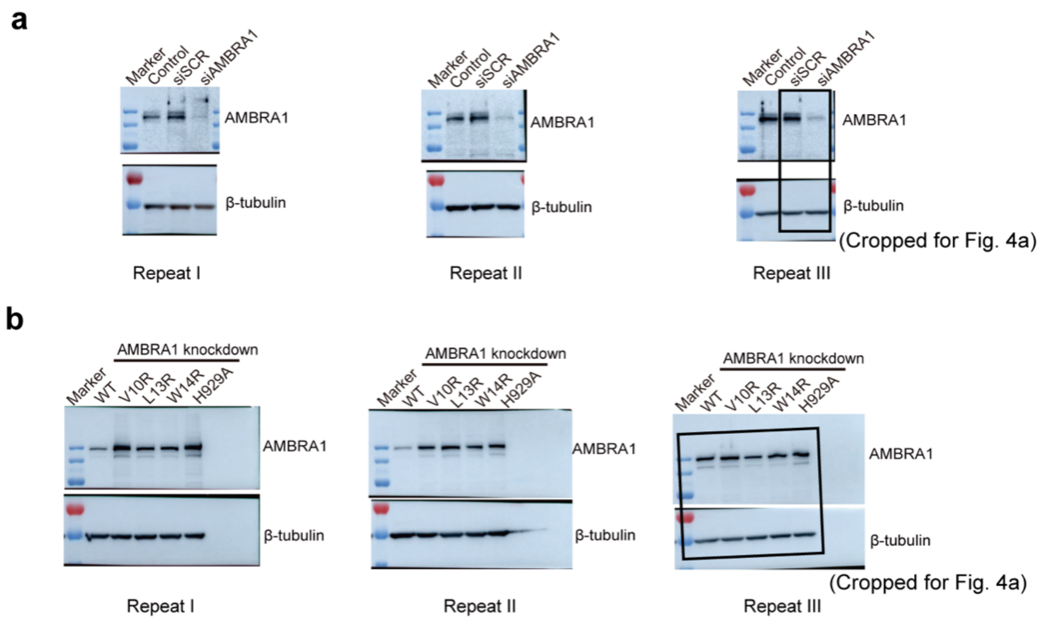

Replicates of AMBRA1 knockdown and single mutants overexpression (related to Fig. 4a)

Supplementary Fig.13: Replicates of AMBRA1 knockdown and re-expression experiments. AMBRA1 downregulation in U2OS cells using specific siRNA oligo ribonucleotide. Wide-type and Single mutants of AMBRA1 were re-expressed in AMBRA1 knockdown cells.

a. AMBRA1 downregulation in U2OS cells using specific siRNA oligo ribonucleotide. AMBRA1 level was analyzed by western blotting, and β-tubulin was used as the protein loading control. Data presented are representative of n = 3 biological repeats.
b. The representative two repeats of Fig. 4a. In AMBRA1 knockdown U2OS cells, re-expression of wide-type and single mutants of AMBRA1. AMBRA1 level was analyzed by western blotting, and β-tubulin was used as the protein loading control. Data presented are representative of n = 3 biological repeats.

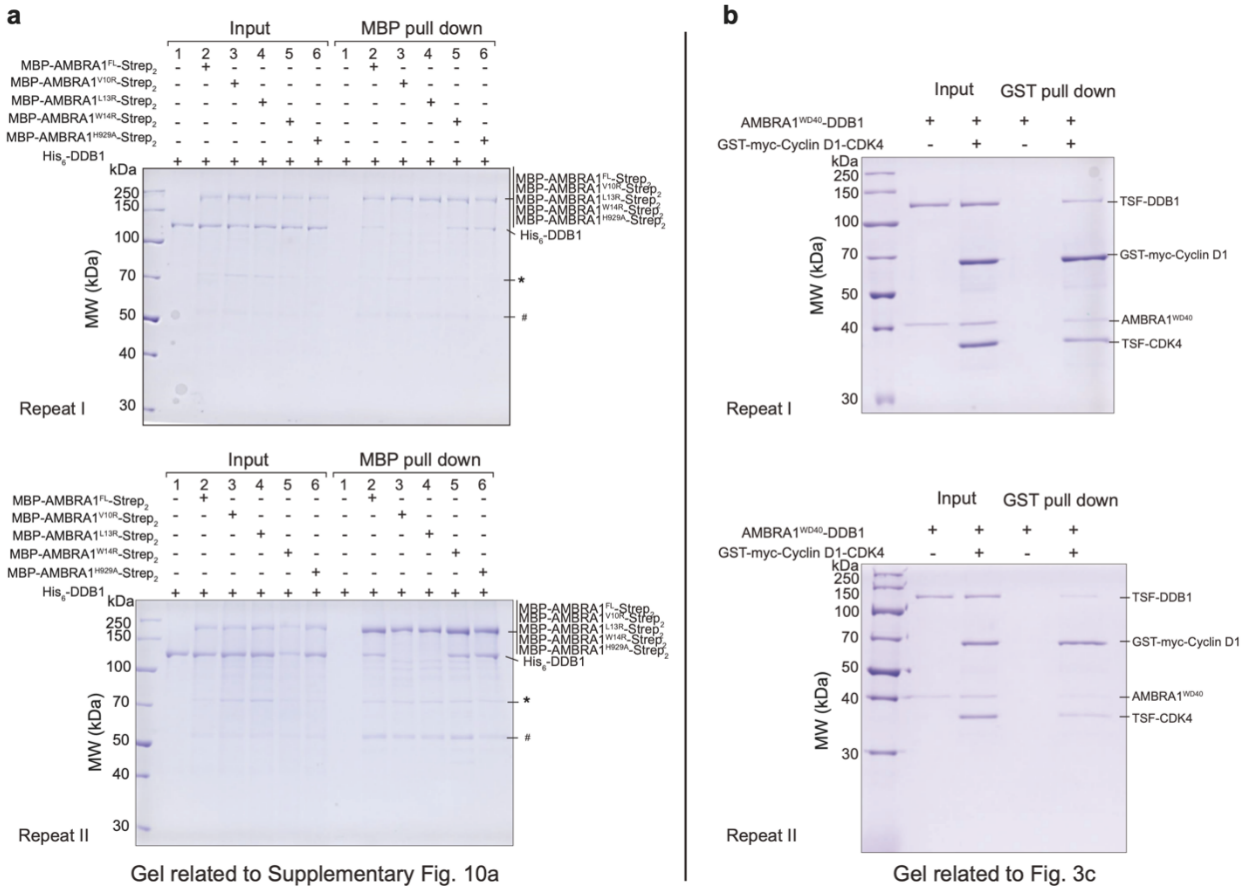

Supplementary Fig.14: Replicates of the MBP and GST pull-down experiments. *In vitro* pull-down experiment of full-length and single mutated AMBRA1 with His_6-tagged DDB1, as well as AMBRA1^{WD40}-DDB1 complex with Cyclin D1-CDK4. The experiment was visualized by SDS–PAGE and Coomassie blue staining. The experiment was repeated at least three times. The asterisk and hash indicate hsp70 and other contaminations.

a. *In vitro* pull-down experiment of the AMBRA1 point mutants with His_6-tagged DDB1. Data presented are representative of n = 3 biological repeats.
b. *In vitro* pull-down experiment of the AMBRA1^{WD40}-DDB1 complex with Cyclin D1-CDK4. Data presented are representative of n = 3 biological repeats.

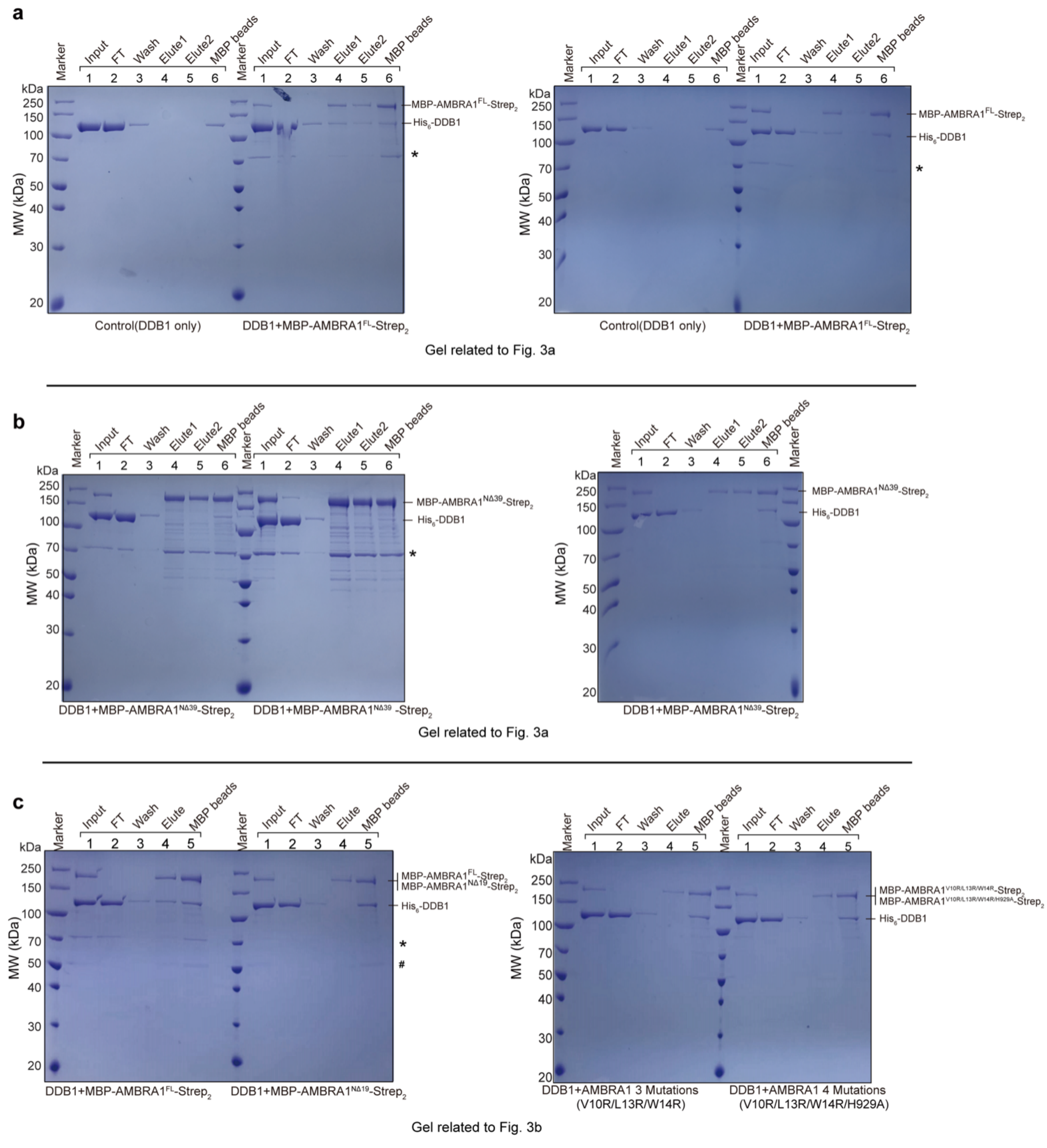

Supplementary Fig.15: Replicates for the MBP pull down experiments. *In vitro* pull-down experiment of full-length, N-terminal 19 residue-deleted, N-terminal 39 residue-deleted and mutated AMBRA1 with His$_6$-tagged DDB1. The experiment was visualized by SDS–PAGE and Coomassie blue staining. FT: Flow through. The asterisk and hash indicate hsp70 and other contaminations.

a. *In vitro* pull-down experiment of the full-length of AMBRA1 with His$_6$-tagged DDB1. Data presented are representative of n = 3 biological repeats.
b. *In vitro* pull-down experiment of the N-terminal 39 residue-deleted of AMBRA1 with His$_6$-tagged DDB1. Data presented are representative of n = 3 biological repeats.
c. *In vitro* pull-down experiment of the N-terminal 19 residue-deleted and mutated of AMBRA1 with His$_6$-tagged DDB1.

Supplementary Table 1. HDX data summary

Data Set	AMBRA1
HDX reaction details	HDX reaction: 90% D_2O, 25°C;<br>D_2O buffer: 20 mM HEPES pH 7.5, 150 mM NaCl, 2 mM $MgCl_2$
HDX time course (sec)	10, 30, 60, 300, 900, 1800
HDX control samples	none
Back-exchange (mean / IQR over entire project)	unknown
# of peptides	203
Sequence coverage	98.84%
Average peptide length / Redundancy	21.59 / 3.38
Replicates	3
Repeatability (avg. stddev of #D)	0.111
Significant differences in HDX (delta HDX > X D - 99% CI)	n/a
Software	HDExaminer 3.3 (Sierra Analytics)

Supplementary Table 2. Cryo-EM data collection, refinement and validation statistics

	DDB1-AMBRA1^{WD40} (EMD-37752)/(PDB 8WQR)
**Data collection and processing**	
Magnification	105,000 X
Voltage (kV)	300 kV
Electron exposure (e–/Å^2)	58.96
Defocus range (μm)	-1 to -1.8
Pixel size (Å)	0.85
Symmetry imposed	C1
Initial particle images (no.)	4,267,168
Final particle images (no.)	780,090
Map resolution (Å)	
FSC threshold	3.08
**Refinement**	
Initial model used (PDB code)	2B5M (DDB1), AMBRA1 from Alphafold2
Model composition	
Non-hydrogen atoms	9675
Protein residues	1414
Ligands	0
B factors (Å^2)	
Protein (min/max/mean)	69.29/405.25/162.27
R.m.s. deviations	
Bond lengths (Å)	0.004
Bond angles (°)	0.763
Validation	
MolProbity score	2.23
Clashscore	16.84
Poor rotamers (%)	0.62
Ramachandran plot	
Favored (%)	91.67
Allowed (%)	8.11
Disallowed (%)	0.22

Supplementary Table 3. Primers used in this study

pCAG-MBP AMBRA1^{FL}-$Strep_2$	Forward primer (5’ to 3’)	CAGGGTGGTACCATGAAGGTGGTGCCTGAG
	Reverse primer (5’ to 3’)	GAATTCCTCGAGTCATCCTTTTTCGAACTGCG GGTGGCTCCACGATCCACCTCCCGATCCACC TCGGGCACCTCCACCTTTCTCGAACTGCGGG TGGCTCCAGCTAGCGCGGTTCCTTGGCTC
pCAG-MBP AMBRA1^{FL}-His_6	Forward primer (5’ to 3’)	CAGGGTGGTACCATGAAGGTGGTGCCTGAG
	Reverse primer (5’ to 3’)	GTGAATTCCTCGAGTCAGTGGTGATGATGGTG ATGGCGGTTC
pCAG-MBP AMBRA1$^{N\Delta39}$-$Strep_2$	Forward primer (5’ to 3’)	CAGGGTGGTACCATGAAGTGGGAGGGGAAAA GAGTGGAAC
	Reverse primer (5’ to 3’)	GAATTCCTCGAGTCATCCTTTTTCGAACTGCG GGTGGCTCCACGATCCACCTCCCGATCCACC TCGGGCACCTCCACCTTTCTCGAACTGCGGG TGGCTCCAGCTAGCGCGGTTCCTTGGCTC
pCAG-MBP AMBRA1$^{N\Delta19}$-$Strep_2$	Forward primer (5’ to 3’)	CAGGGTGGTACCGCTAGGGCTATGGGCGCTC AGAGAC
	Reverse primer (5’ to 3’)	GAATTCCTCGAGTCATCCTTTTTCGAACTGCG GGTGGCTCCACGATCCACCTCCCGATCCACC TCGGGCACCTCCACCTTTCTCGAACTGCGGG TGGCTCCAGCTAGCGCGGTTCCTTGGCTC
pCAG-MBP AMBRA1^{V10R}-$Strep_2$	Forward primer (5’ to 3’)	CAGGGTGGTACCATGAAGGTGGTGCCTGAGA AAAATGCCAGACGCATCCTGTGGGGAAGGGA AAGGGGAGCTAG
	Reverse primer (5’ to 3’)	GAATTCCTCGAGTCATCCTTTTTCGAACTGCG GGTGGCTCCACGATCCACCTCCCGATCCACC TCGGGCACCTCCACCTTTCTCGAACTGCGGG TGGCTCCAGCTAGCGCGGTTCCTTGGCTC
pCAG-MBP AMBRA1^{L13R}-$Strep_2$	Forward primer (5’ to 3’)	CAGGGTGGTACCATGAAGGTGGTGCCTGAGA AAAATGCCGTGCGCATCAGATGGGGAAGGGA AAGGGGAGCTAG
	Reverse primer (5’ to 3’)	GAATTCCTCGAGTCATCCTTTTTCGAACTGCG GGTGGCTCCACGATCCACCTCCCGATCCACC TCGGGCACCTCCACCTTTCTCGAACTGCGGG TGGCTCCAGCTAGCGCGGTTCCTTGGCTC
pCAG-MBP AMBRA1^{W14R}-$Strep_2$	Forward primer (5’ to 3’)	CAGGGTGGTACCATGAAGGTGGTGCCTGAGA AAAATGCCGTGCGCATCCTGAGAGGAAGGGA AAGGGGAGCTAG
	Reverse primer (5’ to 3’)	GAATTCCTCGAGTCATCCTTTTTCGAACTGCG GGTGGCTCCACGATCCACCTCCCGATCCACC TCGGGCACCTCCACCTTTCTCGAACTGCGGG TGGCTCCAGCTAGCGCGGTTCCTTGGCTC
pCAG-MBP AMBRA1$^{V10R/L13R/W14R}$-$Strep_2$	Forward primer (5’ to 3’)	CAGGGTGGTACCATGAAGGTGGTGCCTGAGA AAAATGCCAGACGCATCAGAAGAGGAAGGGA AAGGGGAGCTAG
	Reverse primer (5’ to 3’)	CTAGCTCCCCTTTCCCTTCCTCTTCTGATGCG TCTGGCATTTTTCTCAGGCACCACCTTCATGG TACCACCCTG
pCAG-MBP AMBRA1^{H929A}-$Strep_2$	Middle forward primer (5’ to 3’)	GTGTACAGCCTGGCCCCTGCTAATCTGGGAG AAATGCTG
	Middle reverse primer (5’ to 3’)	CAGCATTTCTCCCAGATTAGCAGGGGCCAGG CTGTACAC
pCAG-GST AMBRA1^{WD40}	Forward primer (5’ to 3’)	CAGGGTGGTACCATGAAGGTGGTGCCTGAGA AAAATG
	Middle forward primer (5’ to 3’)	GCCATCGTGAACCCTTCCAATTCCAACATTGC CAATACTAC
	Middle reverse primer (5’ to 3’)	GTAGTATTGGCAATGTTGGAATTGGAAGGGTT CACGATGGC

	Reverse primer (5' to 3')	GAATTCCTCGAGCTATCCGCTGTTCAGGGCCT CTG
pCAG-GST-Cullin4A	Forward primer (5' to 3')	CTGTATTTTCAGGGTGGTACCATGGCGGACGA GGCCCCGCGGAAG
	Reverse primer (5' to 3')	CTGAGGAGTGAATTCCTCGAGTCAGGCCACG TAGTGGTACTG
pCAG-TSF-Cullin4A	Forward primer (5' to 3')	GATGACGATGACAAGGGTACCATGGCGGACG AGGCCCCGCGGAAG
	Reverse primer (5' to 3')	CTGAGGAGTGAATTCCTCGAGTCAGGCCACG TAGTGGTACTG
pCAG-TSF-DDB1	Forward primer (5' to 3')	GATGACGATGACAAGGGTACCATGTCGTACAA CTACGTGGTAAC
	Reverse primer (5' to 3')	CTGAGGAGTGAATTCCTCGAGCTAATGGATCC GAGTTAGCTCCTC
pCAG-TSF-RBX1	Forward primer (5' to 3')	GACAAGGGTACCATGGCGGCAGCGATGGATG TG
	Reverse primer (5' to 3')	GAATTCCTCGAGCTAGTGCCCATACTTTTGGA ATTC
pCAG-GST-myc-Cyclin D1	Forward primer (5' to 3')	GAAAACCTGTATTTTCAGGGTGAACAAAAACT CATCTCAGAAGAGGATCTGGGTACCATGGAAC ACCAGCTCCTGTGCTG
	Reverse primer (5' to 3')	GTGAATTCCTCGAGTCAGATGTCCACGTCCCG CAC
PCAG-TSF-CDK4	Forward primer (5' to 3')	GATGACGATGACAAGGGTACCATGGCTACCT CTCGATATGAG
	Reverse primer (5' to 3')	CTGAGGAGTGAATTCCTCGAGTCACTCCGGAT TACCTTCATC
pFastBac-Dual-His_6 DDB1	Forward primer (5' to 3')	GGGCGCGGATCCATGCATCACCATCATCACC ACGTCGACGAGGAAAACCTGTACTTCCAGGG CGGAGGCAGGCATATGTCGTACAACTACGTG GTAACG
	Reverse primer (5' to 3')	CAGGCTCTAGACTAATGGATCCGAGTTAGCT

Supplementary References

1. Ma J*, et al.* WDSPdb: an updated resource for WD40 proteins. *Bioinformatics* **35**, 4824-4826 (2019).
2. McGuffin LJ, Bryson K, Jones DT. The PSIPRED protein structure prediction server. *Bioinformatics* **16**, 404-405 (2000).
